# Clinical results of everolimus-eluting stents and sirolimus-eluting stents in patients undergoing percutaneous coronary intervention 

**Published:** 2023

**Authors:** Iraj Jafaripour, Mir Saeid Ramezani, Kamyar Amin, Naghmeh Ziaie Amiri, Mohammad Taghi Hedayati Goudarzi, Fahimeh Elhaminejad

**Affiliations:** 1 Department of Cardiology, Babol University of Medical Sciences, Babol, Iran; 2 Department of Emergency Medicine, Babol University of Medical sciences, Babol, Iran; 3 Babol University of Medical Sciences, Babol, Iran

**Keywords:** Sirolimus-eluting stents, Everolimus-eluting stents, Percutaneous coronary intervention.

## Abstract

**Background:**

It has been pronounced that everolimus-eluting stent (EES) had lower charge of goal-lesion revascularization and stent thrombosis as compared with sirolimus-eluting stents (SES).The goal of this observation was to compare the efficacy and protection of EES with SES in primary percutaneous coronary intervention (PCI) for acute myocardial infarction (AMI).

**Methods:**

In this retrospective study, a total of 404 patients with coronary artery stenosis who underwent angioplasty of one or more coronary arteries were included in the study. Of these, 202 were treated with SES and the others with EES. The data were collected by a questionnaire through which the annual incidence of coronary stent complications including the occurrence of stent thrombosis (confirmed by re-angiography), the occurrence of acute coronary syndrome leading to hospitalization, the occurrence of vascular myocardial infarction related to the stenting vessel, the need for re-angiography and angioplasty and finally the incidence of cardiac mortality were evaluated.

**Results:**

This study showed that the odds ratio of EES thrombosis to SES stent in the unadjusted model is 1.01 (0.06-16.34) and in the adjusted model for confounding variables was equal to 0.80 (0.04-13.35) which in both models, these values were not statistically significant.

**Conclusion:**

The findings of the present study indicate that there is no statistically significant difference between the outcomes in the two groups treated with SES and EES release stents.

The use of stents has appreciably improved the final results of percutaneous coronary interventions (PCI). However, regardless of important advances in angioplasty and stenting, in-stent restenosis and stent thrombosis (1) have remained predominant barriers. From almost two decades in the past, early generation drug-eluting stents (DES) liberating sirolimus (sirolimus-eluting stents [SES]) or paclitaxel-eluting stents [PES] have decreased the need of repeat revascularization as compared with bare-metallic stents and feature emerge as the same old of take care of sufferers undergoing PCI (2-5) despite the fact that the rate of mortality and myocardial infarction (MI) become similar for DES and bare-metal stents (6), very late stent thrombosis emerged as a wonderful entity complication using early generation DES (6). Furthermore, restenosis nevertheless takes place after DES implantation with proof of an erosion of antirestenotic efficacy through the years (7). 

More recent generation DES has been developed with the goal to improve the protection and efficacy of early generation devices (8). The newer generation everolimus-eluting stent (EES) has been shown to improve final results compared with PES (3, 10). However, facts comparing EES with SES are restrained. Due to the fact that SES were shown to be superior in comparison with PES (3, 10). It is relevant to determine whether EES provides therapeutic gain over SES. Due to the increasing access to intervention treatment facilities for more patients and increasing the experience of different treatment centers with these techniques, the study of the effects and consequences of these methods is reasonable.

## Methods

In this retrospective study, a total of 404 patients with coronary artery stenosis who referred to Ayatollah Rouhani Hospital in Babol from March 2014 to March 2019 and underwent angioplasty of one or more coronary arteries were included in the study. Of these, 202 were treated with Sirolimus-eluting stents (Supraflex) and 202 were treated with everolimus-eluting stents (Xience). The study complied with the Declaration of Helsinki and was approved by the Institutional Research Ethics Committee of Babol University of Medical Sciences, Iran (IR.MUBABOL.HRI.REC.1398.016). According to the previous similar study (11) and considering P1 = 0.02, P2 = 0.01, α = 0.05 and β = 0.20, the required sample size was N = 199 in each group.

$\frac{{\left(Z_{1-\frac{\propto }{2}}+Z_{1-\beta }\right)}^{2}(P_{1}\left(1-P_{1}\right)+P_{2}\left(1-P_{2}\right))}{{\left(P_{1}-P_{2}\right)}^{2}}$

Inclusion criteria are; the placement of only one type of stents, including Xience or Supraflex, the patient's willingness and cooperation to participate in the study and completion of the patient questionnaire and medication compliance, and completion of a one-year course of clopidogrel treatment after angioplasty.

 Exclusion criteria are; the simultaneous implantation of other types of stents other than the stents considered in the present study, the simultaneous implantation of both stents in a patient, history of previous angioplasty, presence of concomitant medical diseases with a survival of less than two years, impossibility of referring the patient for periodic post-treatment evaluations, presence of proven drug allergy or resistance to clopidogrel, death of the patient due to non-cardiac and non-coronary causes in the follow-up period.

Data collection was primarily done through re-reading of patients' hospital records and all demographic information of patients and information related to angiography including the number of coronary vessels involved and the number of angioplasty vessels and whether or not had complete revascularization, the presence of risk factors for coronary heart disease through study patients were collected and recorded. 

Secondly, another part of the data was collected by a questionnaire through which the annual incidence of coronary stent complications including the occurrence of stent thrombosis (confirmed by re-angiography), the occurrence of acute coronary syndrome leading to hospitalization, the occurrence of vascular myocardial infarction related to the stenting vessel, the need for re-angiography and angioplasty and finally the incidence of cardiac mortality was evaluated.

Data were analyzed using SPSS software Version 19 (IBM Corporation, Armonk, NY). Then, using descriptive statistics, frequency tables and scattering indices such as mean and standard deviation were obtained. Categorical variables were compared with the chi-square test or Fisher’s exact test. Continuous variables described as mean ± SD were compared by means of student’s t-test. Significance level for all tests in this study was 0.05 and 95% confidence interval was considered.

## Results

A total of 202 patients were assigned to EES, and 202 patients were assigned to SES. The EES and SES groups were well-matched (table 1). This study showed that in terms of the risk of stent thrombosis, statistical analysis consistent with regression (multivariate logistic regression) showed that the odds ratio of EES thrombosis to SES stent in the unadjusted model is 1.01 (0.06-16.34) and in the adjusted model for confounding variables was equal to 0.80 (0.04-13.35) which in both models, these values were not statistically significant. This indicates that there has been no statistically significant difference between EES and SES in terms of risk of stent thrombosis. In terms of the re-admission for cardiac reasons, statistical analysis indicated that the re-admission odds for the EES stents were 0.76 (0.39-1.48) compared to the SES stent in the unadjusted model and 0.76 (0.38-1.53) in the adjusted model for confounding variables. There was no statistically significant difference between EES and SES in terms of the possibility of readmission for cardiac reasons.

As shown in table 2, the probability of needing for angioplasty of the involved vessel was higher in patients in the EES stent group than in the SES stent in the unadjusted model was 2.05 (0.18-22.81) and in the adjusted model for confounding variables was 2.52 (0.20-30.84), which in both models were not statistically significant. Therefore, it is concluded that there is no difference between EES and SES in terms of the possibility of requiring angioplasty of the involved vessel. In this study, considering that the only patient in the group treated with EES stent had myocardial infarction, the calculation of odds of myocardial infarction in both groups was not statistically logical and the small number of events made clinical and statistical conclusions impossible.

The risk of death with all causes in the EES stent group was 4.06 (0.45-36.65) compared to the SES stent in the unadjusted model and 2.48 (0.22-37.64) in the adjusted model for confounding variables. There is no statistically significant difference between the two groups in terms of risk of death due to all causes. 

**Table 1:** Demographic characteristics of patients in two treatment groups

		**EES**	**SES**	**P-value**
**Age (Mean±SD)**		57.45±10.04	58.53±10.75	0.53
**Sex (n, %)**	**Male**	126 (62.4)	131 (64.9)	0.60
	**Female**	76 (37.6)	71 (35.1)	
**Vessels involved (n, %)**	**SVD**	92 (45.6)	98 (48.5)	0.17
	**2VD**	74 (36.6)	59 (29.2)	
	**3VD**	36 (17.8)	45 (22.3)	
**Angioplasty vessels (n, %)**	**One**	189 (93.6)	178 (88.1)	0.10
	**more than one**	13 (6.4)	22 (11.9)	
**Addiction (n, %)**		22 (10.9)	31 (15.3)	0.18
**Smoker (n, %)**		38 (18.9)	44 (21.8)	0.45
**Hypertension (n, %)**		101 (50)	87 (43.1)	0.16
**Diabetes Mellitus (n, %)**		58 (28.7)	49 (24.3)	0.08
**Hyperlipidemia (n, %)**		65 (32.2)	58 (28.7)	0.42
**Chronic kidney disease (n, %)**		2 (1)	1 (0.5)	0.48
**Stroke (n, %)**		6 (3)	8 (4)	0.58
**Normal echocardiography (n, %)**		75 (37.1)	79 (39.1)	0.68
**Number of stents (Mean±SD)**		1.26±0.56	1.29±0.55	0.57
**Stent diameter (Mean±SD)**		2.88±0.34	2.90±0.44	0.51
**Stent length (Mean±SD)**		22.27±8.52	22.01±8.21	0.47

EES= everolimus-eluting stent, SES= sirolimus-eluting stent, SVD=single vessel disease, 2VD=2-vessel disease, 3VD=3-vessel disease, SD=standard deviation.

**Table 2.** Comparison of outcomes in the two groups based on multivariate regression analysis

	**EES** **n (%)**	**SES** **n (%)**	**OR CI(95%)** **crude**	**P**	**OR CI (95%)** **Adj with DM, HLP, CKD, Age, Stent Length**	**P**
**Cardiac readmission**	22 (10.9)	17 (8.4)	0.76 (0.39-1.48)	0.42	0.76 (0.38-1.53)	0.45
**Angioplasty of the affected vessel**	1 (0.49)	2 (1.0)	2.05 (0.18-22.81)	0.55	2.52 (0.20-30.84)	0.46
**Stent thrombosis**	1 (0.49)	1 (0.49)	1.01 (0.06-16.34)	0.99	0.80 (0.04-13.35)	0.87
**Myocardial infarction**	1 (0.49)	-	-	-	-	-
**Cardiac death**	1 (0.49)	4 (19.8)	4.06 (0.45-36.65)	0.17	2.48 (0.22-27.64)	0.45

EES= everolimus-eluting stent, SES= sirolimus-eluting stent, OR=odds ratio, CI=confidence interval, Adj=adjusted, DM=diabetes mellitus, HLP=hyperlipidemia, CKD=chronic kidney disease

## Discussion

The aim of this study was to evaluate and compare the clinical outcomes and major cardiac complications within one year after angioplasty in two groups of PCI patients with stents with EES and SES. In our study, out of 202 patients in the EES group, 10.9% had cardiac readmission, which was less than cardiac hospitalization in the SES group, but after adjusting for risk factors, this difference was not statistically significant. The incidence of definite or probable stent thrombosis in each group was one person per year, which was the same between the two groups. The incidence of heart death in the SES group was slightly higher than in the EES group, which was not statistically significant. 

In a 2017 study by Kandzari et al. (12), procedural success in the SES group was significantly higher. The frequency of in-hospital MI was higher in the EES group than in the SES group. The 30-day target lesion failure was significantly lower in the SES group. There was no statistically difference in mortality between SES group (0.1%) and EES group (0.2%). The rate of revascularization was 0.5% in SES group and 0.7% in EES group. Also, the one-year target lesion failure was 6% in the SES group and 10% in the EES group. More patients had MI target lesion in the EES group than in the SES group, although the mortality rate and cardiac revascularization were equal in both groups. Latent stent thrombosis was significantly lower in the SES group. Compared to the present study, the risk of short-term stent thrombosis was not statistically significant, but patients were not compared for latent thrombosis. Also in the present study, there was no statistically significant difference between the two groups in terms of heart death.

In the study of Han et al. (2018) in terms of device success, the EES group was superior to the SES, but the lesion and procedure success rate were similar in the two groups. It is worth mentioning that baseline cases of TIMI flow III were more in EES group (p <0.05) and post-procedure cases of TIMI flow III were not statistically significant despite being higher in EES group (13). In Park et al.'s (2011) study, cerebrovascular complications were higher in the SES group. In terms of angiography, no difference was observed between the angiographic findings before and after the procedure between the two groups. In general, angiographic findings indicated that the SES-releasing stents were non-inferior to the EES-releasing type. Also, the one-month incidence of clinical complications and the incidence of complications in one-year follow-up were similar in the two groups. There was no statistically significant difference between the two groups in terms of target lesion failure over a period of one year. Although the number of target lesion revascularization was lower in the group of SES-releasing stents, the difference was not statistically significant. Also, the rate of death due to all causes, cardiac death and the incidence of MI in the two groups were not statistically significant (14).

Regarding the relationship between the type of stent used and the risk of stent thrombosis, the findings of the present study indicated that there was no significant difference between the two independent groups. These findings were consistent with the findings of De Winter et al. (2017) (15), Park et al. (2012) (16) but not consistent with the research of Teeuwen et al. (2017) (17), In this study, researchers found that not only were SES-free stents as preventable as EES-free stents or open-segment vein stenosis; these types of stents are more commonly associated with binary restenosis. Regarding the relationship between the type of stent used and the incidence of ACS leading to hospitalization over a period of one year, the findings of the present study indicated that there was no statistically significant difference between the two types of stents. These findings are consistent with the findings of studies conducted by Raber (2011) (18), and Kimura (2012) (19), and it is worth noting that according to the study by Kandzari et al. (2017) (12) all outcomes over a period of one year in patients treated with SES were better than the control group.

Regarding the relationship between the type of stent used and the risk of re-stenosis requiring revascularization in the stented vessel over a period of one year, the results of the present study showed that there was no difference between the types of stents. These findings were confirmed by de Winter et al. (2017) (15) And Park et al. (2012) (16). No statistically significant difference was found between the type of stent used and the risk of myocardial infarction on the stented vessel over a period of one year, which is consistent with studies by Kimura et al. (2012) (19) and Raber et al. (2011) (18). Minimizing secondary complications in patients undergoing intervention is one of the main concerns of physicians in various fields of medicine. Studies suggesting that side effects may occur more frequently with the use of SES-release stents, which has led to a greater tendency to use EES-release stents, which, due to the higher costs associated with these stents, place a greater financial burden on the health care system is imposed. The findings of the present study indicate that there is no statistically significant difference between the outcomes in the two groups treated with SES and EES release stents.
